# Oral and Psychological Alterations in Haemophiliac Patients

**DOI:** 10.3390/biomedicines7020033

**Published:** 2019-04-20

**Authors:** Luca Fiorillo, Rosa De Stefano, Gabriele Cervino, Salvatore Crimi, Alberto Bianchi, Paola Campagna, Alan Scott Herford, Luigi Laino, Marco Cicciù

**Affiliations:** 1 Department of Biomedical and Dental Sciences and Morphological and Functional Imaging, Messina University, 98100 Messina, Italy; lucafiorillo@live.it (L.F.); rsdestefano@libero.it (R.D.S.); gcervino@unime.it (G.C.); 2 Multidisciplinary Department of Medical-Surgical and Odontostomatological Specialties, University of Campania “Luigi Vanvitelli”, 80121 Naples, Italy; luigi.laino@unicampania.it; 3 Department of Surgical and Biomedical Sciences, Catania University, 95123 Catania, Italy; torecrimi@gmail.com (S.C.); alberto.bianchi@unict.it (A.B.); paolacampagna91@gmail.com (P.C.); 4 Department of Maxillofacial Surgery, Loma Linda University, Loma Linda, CA 92354, USA; aherford@llu.edu

**Keywords:** haemophilia, oral alterations, dental risk factor, psychological conditions

## Abstract

Haemophiliacs are hereditary coagulopathies whose basic anomaly consists of the quantitative or qualitative alteration of one or more plasma proteins in the coagulation system. The objective of this review is to analyse all risk factors, predispositions and alterations to the oral-maxillofacial district in patients with haemophilia. The broader assessment also includes the psychological aspects that could affect the treatment and maintenance of oral conditions. The study takes into consideration all the works in the literature in the last 10 years. Works that present oral, dental and psychological changes in haemophilia patients have been combined. A total of 16 studies were analysed carefully evaluating and explaining all the alterations and risk factors that this disease provides. The aim of the review is to report all the anomalies reported in the literature for these patients, and to direct and update the clinician in the treatment of haemophilia patients.

## 1. Introduction

Haemophilia is a disease of genetic origin due to a defect in blood coagulation. It occurs only in the male sex, while women are healthy carriers: this is because it inherits in recessive mode through the X chromosome. Under normal conditions, in the event of leakage from the blood vessels, the blood forms a clot that prevents bleeding. This process involves the chain activation of several plasma proteins. In haemophilia, two of these proteins produced by the liver (factor VIII and factor IX) are deficient or present a functional defect [1,2]. The genes encoding the synthesis of coagulation factors VIII and IX are located on the X chromosome. The X chromosome, carrier of the coagulation defect that determines the haemophilia, is identified as “Xe”. In women, who inherit an X chromosome from each of the two parents, if they have a Xe chromosome, the other non-affected X chromosome will compensate for the production of factor VIII or IX. Males carrying a normal Y chromosome and Xe chromosome are affected by the disease, which is transmitted by the carrier mother (XeY). It is extremely rare for a woman to be affected by haemophilia; in order for this to happen, the father must be affected by haemophilia and the healthy carrier mother. We distinguish mainly two types of haemophilia: haemophilia A is the most common form and is due to a coagulation factor VIII deficiency (incidence is one case for every 10,000 males), while haemophilia B is caused by the lack of factor IX (incidence is one case for every 30,000 males) [3]. Factor VIII is necessary for a rapid conversion of prothrombin into thrombin in native systems. Its activity can be compensated by tissue thromboplastin. Based on the severity of the coagulant factor deficiency, we distinguish three different degrees of haemophilia: the severe form, in which an extremely reduced or absent activity of the coagulant factor (less than 1%) can be demonstrated; the moderate form, in which the plasma levels of factor VIII are between 1% and 5% of normal; and the mild form, in which the plasma levels of factor VIII are between 5% and 40% [1,2,3,4,5,6]. In this paper we will analyse all the correlations present in the literature between the various forms of haemophilia and the psychological conditions of these patients. Furthermore, we will focus on all the oral and dental alterations present. Psychological conditions are closely related to systemic diseases and their assessment of medical treatments is important. Especially for oral, dental and oral surgery treatments it is important to know the conditions of these patients [7,8]. In dentistry, patients are also commonly subjected to surgery under local anaesthesia, so the patient is alert, which is why it is important to know the various psychological facets of these patients.

## 2. Material and Methods

### 2.1. Protocol and Registration

This review is registered at PROSPERO with ID number 122731. PROSPERO is an international database of prospectively registered systematic reviews in health and social care, welfare, public health, education, crime, justice, and international development where there is a health-related outcome. 

### 2.2. Focus Question

The following focus question was developed according to the population, intervention, comparison, and outcome (PICO) study design:

- Is there a correlation between haemophilia and oral and dental alterations? Is their main pathology a risk factor for oral diseases?
- Is there a correlation between haemophilia and psychological conditions?

### 2.3. Information Sources

The search strategy incorporated examinations of electronic databases supplemented by hand searches. A search of four electronic databases, including Ovid MEDLINE, PubMed, and Dentistry, Oral Sciences and Psychology Source, for relevant studies published in the English language from January 2009 to January 2019 was carried out. The search was limited to English language articles. A hand search of the reference lists in the articles retrieved was carried out to source additional relevant publications and to improve the sensitivity of the search.

### 2.4. Search

The keywords used in the search of the selected electronic databases included the following:

- ((((Oral) OR Dental) AND Alterations) OR Defect) AND Haemophilia + Dental Haemophilia.
- ((Psychological AND Conditions) OR Alterations) AND Haemophilia + Psychological Haemophilia.

The keywords were selected to collect and to record as much relevant data as possible without relying on electronic means alone to refine the search results.

### 2.5. Selection of Studies

Two independent reviewers (G.C. and A.S.H.), analysed the obtained papers in order to select inclusion and exclusion criteria as follows. A complete independent dual revision was performed.

### 2.6. Types of Selected Manuscripts

The review included studies on humans published in the English language. Letters, editorials, case reports, and PhD theses were excluded. 

### 2.7. Types of Studies

The review included all human Radomized Controlled Trials (RCTs), prospective and retrospective studies and clinical trials, cohort studies, case–control studies, and case series studies, animal studies and literature review published between January 2009 and January 2019, on haemophilia and dental, oral and psychological alterations.

### 2.8. Disease Definition

#### 2.8.1. Haemophilia

Haemophiliacs are hereditary coagulopathies whose basic anomaly consists of the quantitative or qualitative alteration of one or more plasma proteins in the coagulation system. The entities’ most frequent nosological diseases are represented by: haemophilia A, haemophilia B and the angioemophilia of von Willebrand. Haemophilia A is the most frequent pathology with an incidence estimated at 1:20,000–100,000 male births per year, of which 30% of cases are without familiarity (largely attributable to new mutations). It is a hereditary recessive coagulopathy linked to the X chromosome, in which the male is ill and has variously reduced plasma levels, while the female is usually a healthy carrier. The haemostatic defect derives from the lack of factor VIII to which follows a reduced generation of thrombin through the way intrinsic coagulation. The severity of the clinical picture is related to the degree of deficiency: a normal haemostasis requires a residual activity of> 25%; the majority of patients have levels below 5% and have spontaneous symptoms and complications. The symptomatology is represented by spontaneous haemorrhages or in relation to minor traumas (hematomas, arthropathies, gingivorrhagia, etc.). In 15% of severe haemophiliacs, antibodies of the IgG class (anti-factor VIII inhibitors) appear to decrease the efficacy of substitution therapy and make treatment difficult. Haemophilia B consists of factor IX deficiency and von Willebrand angioemophilia in a joint protein defect plasma of the coagulation cascade and vascular wall; the clinical picture is superimposable and requires replacement therapy with blood products. Based on the severity of the coagulant factor deficiency, we distinguish three different degrees of haemophilia: the severe form, in which an extremely reduced or absent activity of the coagulant factor (less than 1%) can be demonstrated; the moderate form, in which the plasma levels of factor VIII are between 1% and 5% of normal; and the mild form, in which the plasma levels of factor VIII are between 5% and 40%. There is a close correlation between the severity of bleeding symptoms and factor VIII activity levels. The disease in its severe form is characterized by massive haemorrhages, deaths, hemartri and bleeding disproportionate to trauma. In the case of trauma, in fact, the subject can also be at risk of a cerebral haemorrhage but more widespread are the muscular ones, which can cause serious difficulties in movement, and gastrointestinal haemorrhages (hematemesis, melena, proctorragia), bleeding in cavities, haemorrhages of the gold-pharynx, the hemoftoe, epistaxis, hematuria, spinal hematomas. The moderate form is associated with haemorrhagic diathesis which does not lead, if not infrequently, to severe or haemorrhagic bleeding. In cases of mild form, although there may be bleeding after dental avulsions, trauma or after surgery, they are light and never have the character of copious haemorrhages. As for factor IX, this is essential for a prompt conversion of prothrombin into thrombin in native systems, but not in those employing tissue thromboplastin. Clinical manifestations are similar in both forms and only through laboratory tests, or by knowing family history, can we distinguish the two types. For the diagnosis of haemophilia, in the first place, blood analysis is performed to measure the partial thromboplastin time (PTT), which is more prolonged than normal. Confirmation and typing of haemophilia (type A, type B, if it is severe, moderate or mild) is then supported by the deficient plasma protein assay. The treatment consists in the substitution therapy, that is in the administration of the missing factor (factor VIII in haemophilia A, factor IX in haemophilia B) intravenously. The main complication of substitution therapy is the appearance, in the blood of the recipients, of antibodies directed against the factor VIII or IX, called “inhibitors”, which neutralize the effect, and which can make treatment difficult. Patients with mild haemophilia are usually treated with the missing factor concentrate only in case of surgery or after an accident or major injury. However, patients with mild haemophilia can face serious problems, especially because they cannot immediately recognize the signs and symptoms of haemorrhage. This would result in disabling consequences: untreated haemorrhage involves an aggravating pain due to the state of distension to which the surrounding tissues are subjected. In the long run, an articulation affected by a haemorrhage can become arthritic, resulting in chronic pain and disability [1,2,3,4].

#### 2.8.2. Oral Alterations with Genetic Diseases

There are different systemic pathologies, and in particular genetic pathologies that have correlations with oral health. The most striking cases of oral abnormalities are represented by chromosomal pathologies. An example is the Down syndrome, where the patients present anomalies in the oral, hard and soft tissues and teeth. In these pathologies some teeth may be missing, or present anomalies of number or form. Other systemic diseases that show oral and maxillofacial district abnormalities are Gorlin–Golz syndrome, Stevens–Johnson syndrome and Lyell’s syndrome. Kawasaki syndrome also has muco-cutaneous changes and lifonodes. Dental abnormalities are present in Unverricht–Lundborg disease and in juvenile idiopathic arthritis [9,10,11]. Although haemophilia is a hereditary condition, no genetic alterations involving the oral structures are evident. Rather we can talk about risk factors or predisposing factors for the development of oral diseases.

#### 2.8.3. Psychological Aspects of Patients with Systemic Diseases

Surely patients suffering from systemic and genetic diseases have feedback from a psychological point of view. It is possible in some pathologies to highlight proper deficits of a cognitive type. These are associated with organic abnormalities of the patients. As mentioned in the previous paragraph, for example, cognitive impairment is evident in the Down syndrome and in Unverricht–Lundborg disease [11,12]. Cognitive deficits in these patients are not evident, rather their psychological field may have been influenced during growth by the main pathological condition.

#### 2.8.4. Dentistry and Psychology

Dentistry and psychology are closely related. This correlation covers different aspects. Suffice it to say that the term “odontophobia” was coined to describe the fear of the dentist, of dental care or of the dental environment [13]. This paragraph is only a way to quickly mention the correlation between these two fields of medicine. There are also correlations related to the fear of losing teeth or having dental problems. There are problems related to dental aesthetics, not to like oneself. It is certainly important to collaborate during dental care, so as to simplify the work of the clinician. In this article, all the psychological aspects of haemophilia patients were considered, with the aim of making people understand what “psychological” strategies to use during dental treatment [14,15,16].

### 2.9. Inclusion and Exclusion Criteria

The full text of all studies of possible relevance was obtained for assessment against the following inclusion criteria:

- Correlation between oral, dental alterations and haemophilia
- Psychological condition of haemophilic patients

The applied exclusion criteria for studies were as follows:

- Articles published prior to 1 January 2009
- Studies involving patients with other specific diseases
- Not enough information supporting this review
- No access to the title and abstract

### 2.10. Sequential Search Strategy

After a careful analysis of the literature, all the titles of the articles were screened by the authors to eliminate all the non-useful results, case reports and non-English publications. Therefore, the researches were not selected on the basis of data obtained from abstract screening. The last phase involves an analysis of the full text and assesses the eligibility of each study, based on the inclusion and exclusion criteria.

### 2.11. Data Extraction

The data were independently extracted from studies in the form of variables, according to the aims and themes of the present review, as listed onwards.

### 2.12. Data Collections

Data were collected from the included articles and arranged in the following fields (Table 1):

### 2.13. Risk of Bias Assessment

Assessment of risk of bias was undertaken by two authors during data extraction process. For the included studies, this was conducted using the Cochrane Collaboration’s two-part tool for assessing risk of bias [32,33]. An overall risk of bias was then assigned to each trial according to Higgins et al. [33]. The levels of bias were classified as follows: low risk, if all the criteria were met; moderate risk, when only one criterion was missing; high risk, if two or more criteria were missing; and unclear risk, if too few details were making a judgement of certain risk assessment.

## 3. Results

As shown by the studies analysed, there are correlations between haemophilia patients and oral health disorders. Furthermore, there are important correlations between haemophilia patients and psychological conditions. We will broadly evaluate all the various aspects of these correlations later on.

### 3.1. Study Selection

**Figure 1:**
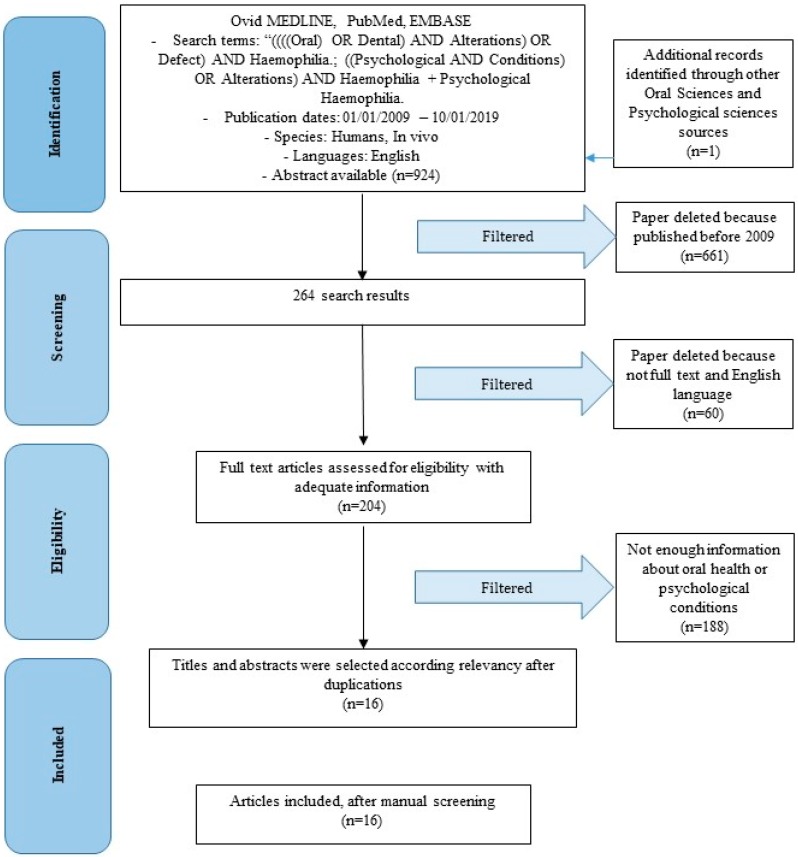
Preferred Reporting Items for Systematic Reviews and Meta-Analyses (PRISMA) flow diagram.

Article review and data extraction were performed according to the Preferred Reporting Items for Systematic Reviews and Meta-Analyses (PRISMA) flow diagram (Figure 1). The initial electronic and hand search retrieved 924 citations and one more paper from Dentistry, Psychological Sciences and Oral Sciences Source with a total of 925 selected papers. After titles and abstracts were reviewed, only 264 articles were published after 1 January 2009, 255 articles were in English, 207 articles were on humans, and only 204 were full-text articles. One hundred and eighty-eight articles were excluded for not having enough information regarding the selected topic, 16 of which were included in this review. Adeyemo et al. [17], in their 2011 study report different oral correlations of haemophiliacs in different forms of the disease. Surely, they report episodes of oral hematomas, palatal purpura or on the tongue, ecchymotic lesions on the lips, furthermore they spontaneously suffer haemorrhages of the tongue and gum. The authors also report a poor quality of hygiene in these patients, with the presence of caries. Cases of post-extractive haemorrhage and hemarthrosis borne by Temporo mandibular Joint (TMJ) are also reported. While all the symptoms mentioned above are present in all forms of haemophilia (A–C), the hemarthroses are not reported in haemophilia C. Unfortunately, the study provided to us by these authors does not provide a sufficient amount of statistical data or percentages for an epidemiological study. On the contrary, another study analysed by Zaliuniene et al. [18] in 2014 provides statistical information. According to the authors, 66% of patients between two and 10 years of age and 73% between seven and 15 years of age appear to be free of caries, compared to only 45% and 41% respectively in healthy patients. Therefore, this data indicates a lower prevalence of caries in haemophiliacs patients. Unfortunately, this study was carried out on a sample from Northern Ireland, and is not always in agreement with other studies. The Decayed Missed Filled Teeth (DMFT) index is also higher in healthy patients rather than in haemophiliacs (2.3 vs 0.45), indicating improved oral health in haemophiliacs. Unfortunately, in other countries the data is not reflected. In Poland or other countries there is a lower level of oral hygiene and a higher DMFT index than the control group, for example this occurs in Egypt and in Pakistan. Others report moderate/severe periodontitis in 80% of haemophilia patients compared to healthy patients (48%). In addition, 30% of patients with mild haemophilia report severe bleeding or spontaneous bleeding or during brushing and this is difficult to manage. Von Willebrand’s disease, haemophilia A and haemophilia B account for 95–97% of all coagulation deficiencies. According to the results, inflammatory diseases of the periodontium, gingivitis and periodontitis are among the most widespread diseases worldwide. About 80–90% of people over the age of 35 are affected. Haemophiliacs, as emerges from the analysis of the articles taken into consideration in this study, present higher gingival indices compared to control groups. Moderate to severe periodontitis was more common among haemophiliacs (80%) than in control groups (48%), all emerging from a radiographic assessment [34].

### 3.2. Study Characteristics

After the selection of the studies all the different aspects regarding the oral anomalies and the cognitive-psychological anomalies were evaluated (Table 1).

- Dental Caries.
- Gengivitis/parodontitis.
- DMFT.
- Hemarthrosis.
- Bleeding.
- Pain.
- Psychological issues.
- Sexual Dysfunction and psychological issues.

### 3.3. Risk of Bias within Studies

Summarizing the risk of bias for each study, most of the studies were classified as unclear risk. More studies were considered as having low risk of bias. Not all the studies analysed in this review of the literature carry useful information to verify the variance of the samples examined. A statistical analysis of variance cannot be performed between studies, but only within other works that present sufficient information to perform statistical calculations. In accordance with the evaluation and the findings of the works, some works present a risk of low bias (since the studies were carried out with a masking of the sample, or other methods such as the double-blind), other works instead present a risk of bias not clear.

### 3.4. Risk of Bias across Studies

The current review includes studies written in English only, which could introduce a publication bias. There were various degrees of heterogeneity in each study design, case selection, and treatment provided among studies.

## 4. Discussion

Dental care tends to be more conservative than in the past and above all thanks to advances in medicine in research it is possible to treat patients suffering from systemic diseases in safety. In this article we wanted to consider all the alterations or risk factor present in the field of oral health in patients affected by various forms of haemophilia. Also considering the psychological aspect, related to care or not. Patients with haemophilia live in a psychological condition of fear, due to what may occur following a trauma or a laceration of the skin or mucous membranes, and therefore the risk of bleeding. Complicating the situation, as evidenced by the articles taken into consideration, is the fact that their susceptibility to pain has increased. Von Willebrand disease, Haemophilia A and Haemophilia B account for 95–97% of all coagulation deficiencies for this reason the topic is current and has a good spread. There is a prevalence of oral diseases in haemophiliacs. The main oral diseases affecting these patients are the same of the rest of population: dental caries and periodontitis or gingivitis. A common alteration in these patients is hemarthras of the temporomandibular joint [17]. According to Zaliuniene et al. [18] patients are afraid to use everyday prophylactic measures in the proper way, such as brushing or flossing, in order to avoid bleeding episodes, and it is conceivable that congenital coagulation disorders are risk of factors for oral pathologies. There is a caries prevalence in both primary and permanent dentitions in children with haemophilia. The DMFT (Decayed Missed Filled Teeth) index was higher in the control than in the haemophilic group. The explanation of such differences could be due to better understanding the importance of periodical attendance for dental treatment. As already mentioned in the previous section, inflammatory diseases of the periodontium, gingivitis and periodontitis involve more haemophiliacs than the control groups [34]. The alterations affecting the oral mucosa, and in particular the inflammatory states [34], can lead to irreversible damage to the periodontium, including damage to alloplastic structures implanted to rehabilitate patients [35]. An aspect to their disadvantage is also represented by rehabilitative surgery, because of the surgical risk on a haemophiliac patient, despite the new bone repair techniques and the new grafting materials promising excellent results [36] According to the guidelines evaluated during this review, it is possible to perform surgery in these patients, though it is even more invasive, but it would be appropriate to limit themselves to strictly necessary surgical practices, thus rehabilitating our patients with alternative methods and not with elective surgeries [3,13]. In some cases, there are clear correlations between neurodegenerative diseases and periodontitis, in this first case there is a difficulty on the part of the patient in performing oral hygiene manoeuvres [37]. In haemophiliac patients, there is a fear of causing bleeding [18]. The majority of the pathologies present in haemophilia patients therefore concern alterations of the DMFT index, and in particular of the caries. However, in these patients there were no anomalies in the structure and shape of the dental elements, so there are no obvious problems with dental tissues such as enamel and dentin. This means that the carious pathologies affecting the dental elements can be treated in a conventional manner, guaranteeing a correct adhesion between the dental materials and the dental elements themselves [38]. Hemarthrosis is a common complication in a haemophiliacs’ weight-bearing joints, yet it rarely occurs in the temporomandibular joint (TMJ). There are few reported cases of TMJ hemarthrosis [17,39,40]. Episodes of spontaneous oral bleeding are reported in the literature in haemophilia patients [41]. Bleeding events may also be induced by poor oral hygiene practices and an iatrogenic factor according to Titilope A. Adeyemo et al. state the locations of oral bleeding are tongue, labial fraenum, buccal mucosa, gingiva and palate. Krüger S et al., in two studies published in international journals, focus on the evaluation of pain perception by these patients. Haemophilic patients suffer from emoarthropathies that lead them to have an increased sensitivity to pain. The somatosensory profile of these patients is impaired [21,22]. A later study by Roussel in 2018 still focused on pain, this study did not focus on joint bleeding as a cause of pain. But it attributes this perception of increased pain to psychological and physiological factors. So, it was a revolutionary study compared to other articles. In patients with haemophilia, therefore, the study of nociceptors and an altered central response to pain should be observed [23]. McLintock assesses the population of women with coagulation disorders and psychological changes [24]. Haemophiliac children are more likely to have psychological problems than their healthy peers. Giving them psychological support can certainly have an important impact on their quality of life. The adult patient with haemophilia instead is forced to adapt his life according to the external world. His work and emotional needs are influenced by the main pathology. Also, in this case, psychological support for the latter is essential [25]. The management of young patients is much debated in different diseases. Unfortunately, in the case of haemophilia, the main pathology is disabling by developing important psychological complications. Alterations in the quality of life and the psychological component have also been highlighted in children with celiac disease [42]. Therefore, haemophiliac patients should be treated as early as possible, as children so as to help them during their growth not to develop psychological problems related to their pathology. Patients suffering from chronic diseases in general are more exposed to psychological pathologies, in this case we must support them [7,26]. Some studies just bring to light the psychological aspect of haemophilia, which can have an important impact on the patient’s life, on its education and on social life as highlighted above [27]. A 2011 study assessed the problems of the sexual sphere in haemophilia patients, focusing on the fact that the clinician should distinguish erectile dysfunction from ejaculatory problems to loss of sexual desire. The therapies in these patients are even more complex due to the risk of comorbidity with other diseases [28]. Basically, by providing care, psychological support to haemophilia patients, it is possible to avoid psychological consequences from an early age [29,30,31]. Other dental aspects, coming back, still concern oral bleeding. This is closely related to the psychological aspect as already mentioned during the article, so as to avoid the manoeuvres of oral prophylaxis (oral hygiene and tooth brushing) to haemophilia patients [20]. Unfortunately, these patients, from a young age often see barriers to their requests for specialized medical treatments such as dental treatment. The refusal by dental practices to be addressed in specialized clinics or hospitals, where the wait times for the treatments are much longer because of haemorrhage risk, further contributes to their fear of dental treatments, this should be considered especially in cases where complex surgeries are to be performed or involving noble structures and at high risk of bleeding. In some cases, moreover, these patients need supportive prophylactic therapy to be able to perform surgical interventions and, as reported in the literature, this is not always possible. Some anti-inflammatory therapies could interfere with platelet aggregation or coagulation as already known. However, the guidelines (as shown in Table 2) for the treatment of these patients are already present in the literature and are not the subject of this study. [19,43,44,45,46,47,48,49,50,51].

There were several limitations present in the current review. This study aims to highlight how this disease may represent a risk factor for oral diseases, and in parallel these patients can develop psychological problems if not properly supported. Much is present in the literature regarding these correlations, but unfortunately there are not many works to support the hemocoagulation pathologies.

## 5. Conclusions

In this paper we have extensively debated the psychological conditions of these patients since their childhood. Unfortunately, this is a disabling pathology that tends to be present in the patients’ daily life. It is undoubtedly a risk factor for the onset of oral diseases, as highlighted during the discussion, these patients, as children, fear the bleeding caused by tooth brushing. This fear, a fear of bleeding that can also be complicated, often leads them to avoid these prophylaxis manoeuvres. Surely this leads to an aggravation of their condition compared to healthy patients. On the other hand, it must be considered that access to dental care by these patients is much more complex, as there are important medico-legal responsibilities in dealing with these patients. Often, colleagues are intimidated by this type of disease and tend to avoid treatments or send them to facilities where waiting times are much longer. We have certainly understood that it is possible to perform surgical practices on these patients by evaluating both systemic and collaborative conditions. however, it is necessary to carry out the appropriate protocols for each patient. As can be seen from some articles taken into account in our review, these patients often have a worsening of oral health conditions, not so much related to their systemic conditions, but due to their difficult management and, from a clinical point of view, and bureaucratic difficulties. Surely the path of research that we started with our team, will move in producing a guideline to the surgical treatment in the dental field of these patients. A guideline that can be used and consulted by all clinicians, so as to be able to treat these patients in a more secure and predictable way. Important support of this work regarding the psychological aspect of these patients, as already highlighted above, is essential during a dental treatment, especially if the patients are still in childhood.

## Figures and Tables

**Table 1:** Alterations highlighted in haemophiliac patients.

Author	Year of Publications	Alterations and Conditions Highlighted
Adeyemo, et al. [17]	2011	Temporo-mandibular Joint (TMJ) hemarthrosis, TMJ pain, Oral district bleeding
Zaliuniene, et al. [18]	2014	Higher DMFT index, inflammatory disorders (gingivitis, periodontitis), bone loss, dental caries, gum health
Segna, et al [19]	2017	Oral district bleeding
Kumar, et al. [20]	2013	Oral district bleeding
Krüger, et al. [21]	2018	Enhanced pain sensitivity
Krüger, et al. [22]	2018	Alterations of the somatosensory profile
Roussel, et al. [23]	2018	Nociceptive alterations
McLintock, et al. [24]	2018	Psychological issues in women with Bleeding Disorders
Limperg, et al. [25]	2017	Psychosocial risk
Torres-Ortuño, et al. [7]	2017	Psychological issues
García-Dasí, et al. [26]	2016	Psychological issues
Giordano, et al. [27]	2014	Psychological issues
Cassis, et al. [28]	2012	Psychological issues and quality of life
Bar-Chama, et al. [29]	2011	Psychological issues and sexual dysfunctions
Coppola, et al. [30]	2011	Psychological issues
Mauser-Bunschoten, et al. [31]	2009	Psychosocial issues during ageing

“Author (Year)”—revealed the author and year of publication; “Oral alterations”—described types oral alterations or risk factors highlighted; “Psychological conditions”—psychological conditions of the patients.

**Table 2:** Nelson et al. [43] Surgical teeth extraction guideline.

Steps	Instructions
1	Dental extraction or surgical procedures carried out within the oral cavity should be performed with a plan for haemostasis management, in consultation with the haematologist.
2	Tranexamic acid or epsilon aminocaproic acid (EACA) is often used after dental procedures to reduce the need for replacement therapy.
3	Oral antibiotics should only be prescribed if clinically necessary.
4	Local haemostatic measures may also be used whenever possible following a dental extraction. Typical products include oxidized cellulose and fibrin glue.
5	Following a tooth extraction, the patient should be advised to avoid hot food and drinks until normal feeling has returned. Smoking should be avoided as this can cause problems with healing. Regular warm salt water mouthwashes (a teaspoon of salt in a glass of warm water) should begin the day after treatment and continue for 5–7 days or until the mouth has healed.
6	Prolonged bleeding and/or difficulty in speaking, swallowing, or breathing following dental manipulation should be reported to the haematologist/dental surgeon immediately.
7	Non-steroidal anti-inflammatory drugs (NSAIDs) and aspirin must be avoided.
8	An appropriate dose of paracetamol/acetaminophen every 6 h for 2–3 days will help prevent pain following an extraction.
9	The presence of blood-borne infections should not affect the availability of dental treatment.
10	Prevention of bleeding at the time of dental procedures in patients with inhibitors to FVIII or FIX requires careful planning.
